# Exogenous Cardiac Bridging Integrator 1 Benefits Mouse Hearts With Pre-existing Pressure Overload-Induced Heart Failure

**DOI:** 10.3389/fphys.2020.00708

**Published:** 2020-06-24

**Authors:** Jing Li, Sosse Agvanian, Kang Zhou, Robin M. Shaw, TingTing Hong

**Affiliations:** ^1^Department of Cardiology, Smidt Heart Institute, Cedars-Sinai Medical Center, Los Angeles, CA, United States; ^2^Nora Eccles Harrison Cardiovascular Research and Training Institute, University of Utah, Salt Lake City, UT, United States; ^3^Department of Pharmacology & Toxicology, College of Pharmacy, University of Utah, Salt Lake City, UT, United States

**Keywords:** heart failure, ion channel, cardiac bridging integrator 1, calcium handling, gene therapy

## Abstract

**Background**: Cardiac bridging integrator 1 (cBIN1) organizes transverse tubule (t-tubule) membrane calcium handling microdomains required for normal beat-to-beat contractility. cBIN1 is transcriptionally reduced in heart failure (HF). We recently found that cBIN1 pretreatment can limit HF development in stressed mice. Here, we aim to explore whether cBIN1 replacement therapy can improve myocardial function in continuously stressed hearts with pre-existing HF.

**Methods**: Adult male mice were subjected to sham or transverse aortic constriction (TAC) surgery at the age of 8–10 weeks old. Adeno-associated virus 9 (AAV9) transducing cBIN1-V5 or GFP-V5 (3 × 10^10^ vg) was administered through retro-orbital injection at 5 weeks post-TAC. Mice were followed by echocardiography to monitor cardiac function until 20 weeks after TAC. Overall survival, heart and lung weight (LW), and HF incidence were determined. In a second set of animals in which AAV9-cBIN1 pretreatment prevents HF, we recorded cardiac pressure-volume (PV) loops and obtained myocardial immunofluorescence imaging.

**Results**: The overall Kaplan-Meir survival of AAV9-cBIN1 mice was 77.8%, indicating a significant partial rescue between AAV9-GFP (58.8%) and sham (100%) treated mice. In mice with ejection fraction (EF) ≥30% prior to AAV9 injection at 5 weeks post-TAC, AAV9-cBIN1 significantly increased survival to 93.3%, compared to 62.5% survival for AAV9-GFP treated mice. The effect of exogenous cBIN1 was to attenuate TAC-induced left ventricular (LV) dilation and prevent further HF development. Recovery of EF also occurs in AAV9-cBIN1-treated mice. We found that EF increases to a peak at 6–8 weeks post-viral injection. Furthermore, PV loop analysis identified that AAV9-cBIN1 increases both systolic and diastolic function of the post-TAC hearts. At the myocyte level, AAV9-cBIN1 normalizes cBIN1 expression, t-tubule membrane intensity, and intracellular distribution of Cav1.2 and ryanodine receptors (RyRs).

**Conclusions**: In mice with pre-existing HF, exogenous cBIN1 can normalize t-tubule calcium handling microdomains, limit HF progression, rescue cardiac function, and improve survival.

## Introduction

Heart failure (HF) is the fastest growing cardiovascular disorder affecting over 20 million people worldwide and 6.2 million Americans (Roger, 2013; Virani et al., 2020). The majority of HF-related mortality is associated with cardiac pump failure due to myocardial inotropic and lusitropic dysfunction, as well as sudden cardiac death due to increased arrhythmia burden of failing hearts. Furthermore, near 50% of HF patients are diagnosed with HF with preserved ejection fraction (HFpEF), which develops severe diastolic failure, has increased risk of arrhythmias, and lacks effective medical therapy (Virani et al., 2020). Thus, there is an urgent need to develop new therapeutic strategies that can limit and reverse HF progression.

During HF development, the pathophysiologic cellular hallmark of failing ventricular myocytes is abnormal calcium transients with impaired intracellular calcium homeostasis (Gomez et al., 1997, 2001; Litwin et al., 2000; Louch et al., 2004), which disrupts excitation-contraction (EC) coupling (Perreault et al., 1992), impairs electrical stability (Landstrom et al., 2017), and disturbs mitochondrial metabolism (Lopez-Crisosto et al., 2017). Normal beat-to-beat calcium transient relies on a sequence of intracellular events known as calcium-induced-calcium-release (CICR; Bers, 2002), where t-tubule L-type calcium channels (LTCCs)-mediated initial calcium influx will subsequently induce a massive calcium release *via* ryanodine receptors (RyRs) from the sarcoplasmic reticulum (SR) store. During relaxation, the accumulated calcium will then be removed from the cytoplasm mainly by calcium reuptake to SR *via* SR Ca^2+^-ATPase 2a (SERCA2a) together with calcium exclusion through sodium calcium exchanger from cytosol into the extracellular space (Bers, 2008). In HF, abnormal t-tubule remodeling (Lyon et al., 2009; Louch et al., 2010; Wei et al., 2010) impairs LTCC-RyR coupling and synchronous CICR (Gomez et al., 1997; Litwin et al., 2000), resulting in diminished systolic release, EC uncoupling, and thus reduced contractility. On the other hand, HF-associated leaky RyRs (Marx et al., 2000) and abnormal SERCA2a function (Houser et al., 2000) will result in SR depletion and elevated diastolic calcium (Periasamy and Huke, 2001), resulting in severe diastolic failure and electrical instability (Erkasap, 2007). In addition, impaired calcium homeostasis triggers loss of mitochondrial membrane potential (Santulli et al., 2015) and increased permeability (Odagiri et al., 2009), which promotes the risk of mitochondrial-initiated cell death (Nakayama et al., 2007; Kinnally et al., 2011) and HF progression (Nakayama et al., 2007; Zhou and Tian, 2018). Taken together, calcium homeostasis is critical in maintaining normal cardiac pump function, electrical stability, and metabolism. Disturbed beat-to-beat calcium dynamic, as occurs in diseased hearts, will therefore lead to pump failure, lethal arrhythmias, and severe metabolic disorder.

Recently, we reported that the reorganization of intracellular calcium handling machinery could be achieved by a new approach of targeting t-tubule membrane microdomains organized by the cardiac bridging integrator 1 (cBIN1; Liu et al., 2020). We previously found that BIN1 facilitates intracellular LTCC trafficking to t-tubule microdomains (Hong et al., 2010), as well as surface clustering (Fu et al., 2016; Fu and Hong, 2016) at the t-tubule microdomains. RyRs are recruited to junctional SR (jSR) by cBIN1 for coupling with LTCCs (Fu et al., 2016). In addition to dyad organization, cBIN1 sculpted microdomains generate a protective slow diffusion zone for extracellular ions in t-tubule lumen to regulate ionic flux across t-tubule membrane (Hong et al., 2014). More recently, we found that cBIN1-microdomain is also critical in organizing the intracellular distribution of SERCA2a for diastolic calcium regulation (Liu et al., 2020). In HF, cBIN1-microdomains are disrupted due to transcriptional reduction in cBIN1 (Hong et al., 2012b; Caldwell et al., 2014; Zhou and Hong, 2017), impairing dyad formation, calcium transient regulation, and cardiac contractility. Reduced myocardial cBIN1 can be detected in human blood, a result of cBIN1-membrane turnover and microparticle release (Xu et al., 2017). In humans, plasma CS (cBIN1 score) is an index of myocyte cBIN1 level, which identifies myocardial structural remodeling, facilitating HF diagnosis and prognosis (Nikolova et al., 2018). In mouse hearts subjected to chronic stress, pretreatment with exogenous cBIN1 preserves the microdomain-organized distribution of Cav1.2 and SERCA2a, maintaining normal inotropy and lusitropy (Liu et al., 2020). These data indicate that cBIN1 replacement can be an effective HF therapy with the potential to recover myocardial function in hearts with preexisting HF.

Since increased afterload is an important primary and secondary cause of HF (Blaufarb and Sonnenblick, 1996), our current study uses a mouse model of elevated afterload induced by transverse-aortic constriction (TAC). In TAC mice, we recently reported that cBIN1 pretreatment prevents HF development (Liu et al., 2020). Here, we used adeno-associated virus 9 (AAV9)-mediated gene transfer to introduce exogenous cBIN1 in post-TAC mouse hearts with pre-existing HF. We find that *cBin1* gene therapy reduces TAC-induced pathological remodeling, limits HF progression, causes functional recovery, and ultimately reduces death and improves survival.

## Materials and Methods

### Animal Model Design

All mouse procedures were reviewed and approved by the Cedars-Sinai Medical Center (CSMC) and University of Utah Institutional Animal Care and Use Committees (IACUC) and conform to the Guide for the Care and Use of Laboratory Animals published by the U.S. National Institutes of Health (NIH Publication No. 85–23, revised 2011).

Adult male C57BL/6 mice (The Jackson Laboratory) were used. All mice were anesthetized at the age of 8–10 weeks and subjected to open-chest sham or TAC surgery. TAC was performed by tying a 7-0 silk suture against a 27-gauge needle between the first and second branch of the aortic arch. For sham controls, age-matched mice were subjected to open-chest mock surgery without TAC being performed. For rescue experiments, at 5 weeks post the onset of TAC, mice received retro-orbital injection of 100 μl of 3 × 10^10^ vector genome (vg) of AAV9 virus (Welgen, Inc.) transducing cBIN1-V5 or GFP-V5 (Basheer et al., 2017). In the prevention experiments where cBIN1-V5 was reported to provide protection of mouse hearts against subsequent TAC-induced HF (Liu et al., 2020), mice received the same dose (3 × 10^10^ vg) of AAV9 virus transducing cBIN1-V5 and GFP-V5 3 weeks before TAC surgery was done.

### Echocardiography

*In vivo* systolic and diastolic left ventricular (LV) functions were monitored by echocardiography in anesthetized mice using Vevo 3100 at baseline, pre-surgery, and every other week thereafter until the end of the experimental protocol. The trans-aortic pressure gradient was recorded using the modified Bernoulli equation [Δ Pressure gradient (mmHg) = 4 × peak velocity^2^ (m/s)^2^] at 2 weeks post-surgery. All surviving mice at 5 weeks post-TAC were included in the study.

### Hemodynamic Measurements

In the prevention experiments, 8 weeks after TAC, mice were anesthetized with 3% isoflurane in 100% O_2_ at 1.2 L/min and maintained at 1.5% isoflurane during the measurement. After intubation to facilitate breathing, mice were placed on controlled heating pads maintained at 37°C. A 1-Fr pressure-conductance microcatheter (PVR-1045; Millar Instruments, Houston, TX) was inserted into the right carotid artery and advanced into the LV. After stabilization for 10 min, baseline P-V relations were recorded. Signals were continuously recorded using a P-V conductance system (MPVS-Ultra; Millar Instruments) connected to the PowerLab data acquisition system (AD Instruments, Colorado Springs, CO), stored, and displayed on a PC computer by the LabChart7 Software System (AD Instruments). With the use of a special P-V analysis program (PVAN; Millar Instruments), heart rate, the maximal slope of LV systolic pressure increment (dp/dt max) and diastolic pressure decrement (dp/dt min), EF, and Tau value were recorded and calculated as previously described (Pacher et al., 2008).

### Immunofluorescence Labeling and Confocal Imaging

For cardiomyocyte membrane fluorescent labeling, freshly isolated ventricular cardiomyocytes from GFP-TAC and cBIN1-TAC mice were incubated with Di-8-ANNEPs for 20 min at room temperature (RT). The cells were then washed with HBSS to remove the remaining dye before live-cell imaging. For fixed-cell V5 imaging (10×), isolated cardiomyocytes were fixed in methanol at −20°C for 5 min and permeabilized and blocked with 0.5% Triton X-100 and 5% normal goat serum (NGS) in PBS for 1 h at RT. Cells were incubated with rabbit anti-V5 (Sigma) overnight at 4°C and detected by Alexa555 conjugated goat anti rabbit IgG. For tissue immunofluorescent imaging, myocardial cryo-sections were fixed with ice-cold acetone for 5 min. The primary antibodies used were mouse anti–BIN1-BAR (2F11, Rockland), mouse anti-RyR (Abcam), or rabbit anti-Cav1.2 (Alomone). Following incubation with primary antibodies and several washes with 1× PBS, cells and tissue sections were then incubated with Alexa488 or Alexa555 conjugated goat anti-mouse or rabbit secondary antibodies (Life Technologies) and mounted with DAPI containing ProLong gold.

All confocal imaging was performed on a Nikon Eclipse Ti microscope with a 100 × 1.49 numerical aperture (NA) and 60 × 1.1 or 10 × objectives. High-resolution cardiomyocyte images were obtained using a spinning-disc confocal unit (Yokogawa CSU10) with diode-pumped solid state (DPSS) lasers (486, 561, 647) generated from laser merge module 5 (Spectral applied research, CA). T-tubule membrane labeling fluorescent intensity profiles were generated by ImageJ, and peak intensity at t-tubules is quantified as previously reported (Hong et al., 2014). Power spectrum analysis was analyzed in Matlab using FFT conversion, and normalized peak power density at t-tubules was compared across groups (Hong et al., 2010; Wei et al., 2010).

### Western Blotting

Tissue lysates were made from hearts flash frozen in liquid nitrogen. Frozen tissue was homogenized in radio-immunoprecipitation assay (RIPA) lysis buffer as previously described (Liu et al., 2020). Lysates were rotated head-to-toe in 4°C for 40 min, sonicated, followed by centrifugation (16,000 *g* for 25 min at 4°C) to clear cellular debris. Protein lysates were then prepared 2× sample buffer (Bio-Rad, Hercules, CA) containing 5% β-mercaptoethanol, incubated in RT for 30 min, and separated on an 8–12% gradient sodium dodecyl sulfate (SDS) polyacrylamide electrophoresis gel. Proteins were electro-transferred to polyvinylidene difluoride (PVDF) membrane. After transfer, membranes were fixed in methanol and blocked with 5% BSA in 1× tris-buffered saline (TBS) for 1 h at RT, and incubated with primary antibody in 5% BSA in 1× TBS overnight at 4°C, followed by incubation with Alexa 647 conjugated secondary antibody (Life Technology) for 1 h at RT. Primary antibodies consisted of a custom-made polyclonal rabbit anti-BIN1 exon 13 (Anaspec) (Hong et al., 2014), mouse anti RyR (Abcam), rabbit Cav1.2 antibody (Alomone), and mouse anti-GAPDH (Millipore).

### Statistical Analysis

All data are expressed as mean ± standard error of the mean (SEM). Normality was assessed using the Shapiro-Wilk test. Kaplan-Meier survival analysis was used to compare survival between two or three groups using the long-rank test. Continuous variables were compared using Mann-Whitney U and one-way ANOVA or Kruskal-Wallis tests. Two-way ANOVA followed by LSD *post-hoc* test was used to determine differences between two groups at two different time points. Categorical variables were analyzed using Fisher’s exact or Chi-square tests. Data were analyzed using GraphPad Prism 7.0. Two-sided *p* values were used and *p* < 0.05 is considered statistically significant.

## Results

**Figure 1:**
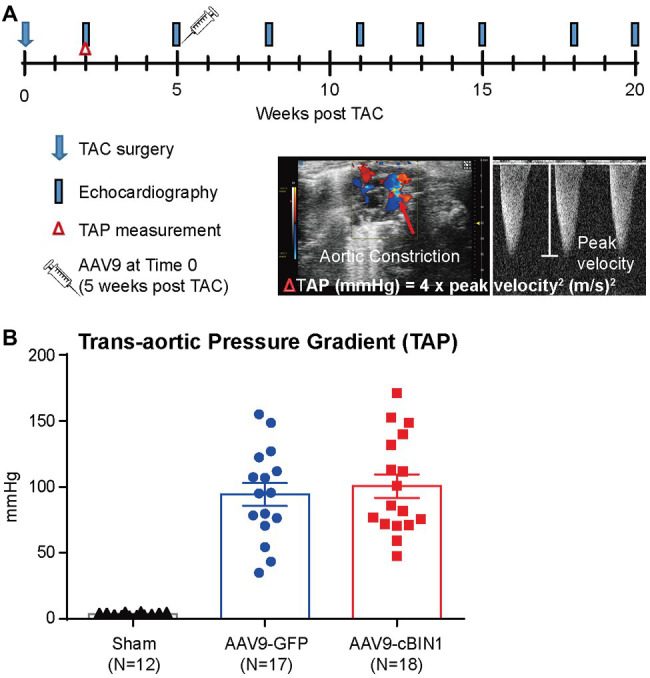
Experimental protocol of cardiac bridging integrator 1 (cBIN1) post-treatment in mice subjected to transverse aortic constriction (TAC). **(A)** Schematic protocol: 47 mice were randomized into three groups: sham (*N* = 12) or TAC mice with post-treatment of AAV9-GFP (*N* = 17) and AAV9-cBIN1 (*N* = 18) administered at 5 weeks post-TAC. **(B)** Echocardiography analysis of trans-aortic pressure gradient (TAP) in the three groups.

To explore whether targeting cBIN1-microdomain can be a new therapy for HF, we investigated how cardiac cBIN1 affects HF development in mice subjected to pressure overload stress. As indicated in the experimental protocol in Figure 1A, mice were subjected to TAC first for 5 weeks before retro-orbital injection of AAV9 transducing cBIN1-V5 or control GFP-V5, followed by echocardiography monitoring for an additional 15 weeks after virus injection (end point at 20 weeks post-TAC). In addition to a group of mice subjected to an open-chest mock surgery (sham control, *N* = 12), 36 mice were subjected to TAC surgery. One mouse died before reaching Time 0 (5 weeks post-TAC), the remaining 35 surviving mice were randomized to receive AAV9-GFP (*N* = 17) or cBIN1 (*N* = 18) at 3 × 10^10^ vg. Anti-V5 labeling of cardiomyocytes isolated from mice after 15 weeks of AAV9 injection identified positive V5 signal, indicating successful transduction of exogenous protein in cardiomyocytes (Supplementary Figure 1). Comparable trans-aortic pressure gradient at 2 weeks post-TAC (Figure 1B) and myocardial dysfunction at 5 weeks post-TAC in these two groups (Table 1) establish a similar level of TAC-generated pressure overload and its induction of dilated cardiomyopathy.

**Table 1:** Echocardiographic and physiological parameters of AAV9-GFP or AAV9-cBIN1 injected mice before and after AAV9 injection.

	Pre-AAV9 (5w-post TAC)			Post-AAV9 (20w-post TAC)		
	SHAM	GFP	cBIN1	SHAM	GFP	cBIN1
EF (%)	53.72 ± 2.01	41.87 ± 3.20^**^	44.22 ± 2.83^*^	48.25 ± 2.22	27.65 ± 4.19^***^	41.72 ± 1.72^##^
LVEDV (μl)	62.34 ± 3.87	82.35 ± 5.32^*^	78.48 ± 6.22	58.24 ± 2.52	107.64 ± 10.01^***^	77.96 ± 4.55^##^
LVESV (μl)	28.97 ± 2.46	49.44 ± 4.96^*^	46.43 ± 5.89^*^	35.29 ± 2.03	79.92 ± 10.43^***^	45.76 ± 3.43^###^
HR (bpm)	482.04 ± 14.76	516.60 ± 8.49	481.41 ± 12.40	463.77 ± 12.28	557.35 ± 20.71^***^	504.94 ± 8.91^*^^,^ ^##^
SV (μl)	33.38 ± 2.17	32.91 ± 2.38	32.05 ± 1.19	32.95 ± 1.98	27.72 ± 3.28	32.20 ± 1.79
CO (ml/min)	16.00 ± 1.08	16.92 ± 1.18	15.30 ± 0.53	15.35 ± 1.17	15.37 ± 1.83	16.34 ± 1.08
LVAWs (mm)	1.40 ± 0.04	1.61 ± 0.07^*^	1.56 ± 0.05	1.35 ± 0.08	1.52 ± 0.08	1.61 ± 0.06^*^
LVAWd (mm)	0.99 ± 0.03	1.26 ± 0.06^***^	1.20 ± 0.03^**^	0.97 ± 0.06	1.29 ± 0.07^***^	1.28 ± 0.05^***^
LVPWs (mm)	1.15 ± 0.07	1.46 ± 0.07^**^	1.34 ± 0.06	1.10 ± 0.03	1.41 ± 0.15^*^	1.33 ± 0.07
LVPWd (mm)	0.79 ± 0.05	1.15 ± 0.07^***^	1.06 ± 0.04^**^	0.84 ± 0.03	1.24 ± 0.16^***^	1.11 ± 0.06^*^
LV Mass (mm)	124.39 ± 4.76	232.36 ± 15.20^***^	202.30 ± 8.43^***^	137.17 ± 9.60	300.76 ± 29.55^***^	223.09 ± 12.41^***^^,^ ^###^
BW (g)	27.83 ± 0.65	29.66 ± 0.56	29.28 ± 0.40	32.41 ± 1.19	33.97 ± 0.84	34.77 ± 0.95^*^
HW (mg)	-	-	-	196.98 ± 7.25	288.63 ± 31.63^***^	234.94 ± 10.75^#^
LW (mg)	-	-	-	158.32 ± 5.14	245.70 ± 63.18^***^	165.28 ± 3.68^#^
HW/TL (g/m)	-	-	-	9.85 ± 0.36	14.77 ± 1.40^***^	11.75 ± 0.54^#^
LW/TL (g/m)	-	-	-	7.92 ± 0.26	12.53 ± 3.09^*^	8.26 ± 0.18

EF, ejection fraction; LVEDV, left ventricular end-diastolic volume; LVESV, left ventricular end-systolic volume; HR, heart rate; SV, stroke volume; CO, cardiac output; LVAWs/LVAWd, left ventricular anterior wall in systole/diastole; LVPWs/d, left ventricular posterior wall in systole/diastole; LV Mass, left ventricular mass; BW, body weight; HW, heart weight; LW, lung weight; TL, tibia length.

Data are expressed as mean ± SEM.

* Indicates *p* < 0.05.

** Indicates *p* < 0.01.

*** Indicates *p* < 0.001 vs. sham.

# Indicates *p* < 0.05.

## Indicates *p* < 0.01.

### Indicates *p* < 0.001 for AAV9-GFP vs. AAV9-cBIN1.

**Figure 2:**
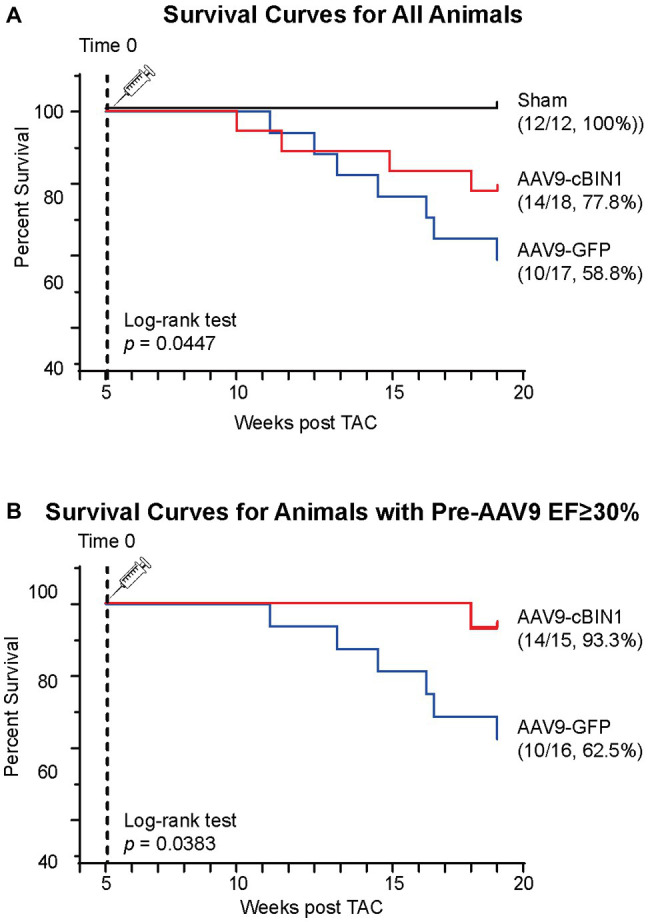
Post-treatment with exogenous cBIN1 improves survival rates in post-TAC mice. **(A)** Kaplan-Meier survival curves for all three groups of mice: sham controls (*N* = 12), post-TAC mice treated with AAV9-CMV viruses transducing GFP (*N* = 17) or cBIN1 (*N* = 18). The log-rank test was used for comparison across three groups. **(B)** Kaplan-Meier survival curves for post-TAC mice not yet at end stage of disease prior to AAV9 injection (5 weeks post-TAC EF ≥ 30%) following post-treatment of AAV9-GFP (*N* = 16) or AAV9-cBIN1 (*N* = 15). The log-rank test was used for comparison of survival rates between AAV9-GFP and AAV9-cBIN1 groups.

**Figure 3:**
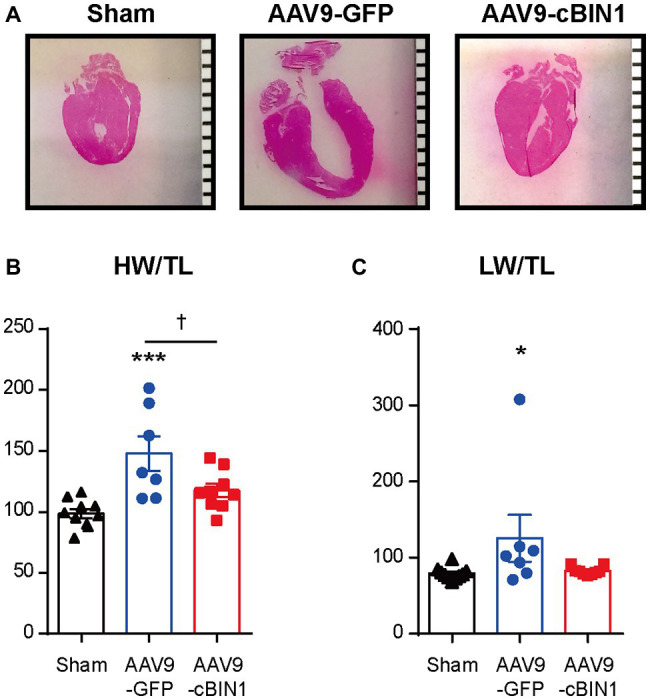
Exogenous cBIN1 reduces TAC-induced hypertrophy and pulmonary edema. **(A)** Longitudinal heart sections with H&E staining, (scale bar, 1 mm). **(B)** The ratio of heart weight over tibia length (HW/TL) and **(C)** lung weight over tibia length (LW/TL) at 20 weeks post-TAC. Data are presented as mean ± SEM, and two-way ANOVA with Fisher’s LSD test was used for statistical analysis. ^*,***^p < 0.05, 0.001 vs. sham; ^†^*p* < 0.05, comparing between GFP and cBIN1 groups.

We, then, explored the overall survival rate (non-survival is death) in all groups. As indicated in the Kaplan-Meier curves in Figure 2, the survival curve of AAV9-cBIN1 treated TAC mice (survival rate 77.8%, 14/18) lies significantly between the two curves of sham control (survival rate 100%, 12/12) and AAV9-GFP treated TAC mice (survival rate 58.8%, 10/17; *p* = 0.045 by log-rank test when comparing the three groups; Figure 2A), indicating partial rescue. Next, in all TAC mice not yet at end stage disease with EF ≥ 30% at Time 0 (5 weeks post-TAC and before virus injection), we further compared the survival rates between the two viral groups. The survival rate was significantly improved by AAV9-cBIN1 (*p* = 0.038 by log-rank test) when compared to the AAV9-GFP group (Figure 2B). Of the 16 AAV9-GFP mice with EF ≥ 30% at Time 0, six died within 20 weeks post-TAC with progressive EF reduction. In comparison, of the 15 AAV9-cBIN1 mice with EF ≥ 30% at Time 0, only one died within 20 weeks post-TAC. Note, all four animals from both groups with pre-AAV9 EF < 30% died during follow up, suggesting that AAV9-cBIN1 is not sufficient to improve survival in animals already at end-stage HF and with afterload still restricted. The surviving mice at 20 weeks post-TAC were then sacrificed for tissue collection for histological analysis (Figure 3A). Compared to the normal structure and size of the sham control hearts, AAV9-GFP hearts were visibly enlarged with an expanded left ventricle, which was reduced in AAV9-cBIN1 hearts. The ratios of heart weight (HW) and lung weight (LW) over tibial length (TL) were further evaluated. AAV9-cBIN1 mice did not have a significant increase in HW/TL and LW/TL as occurred in AAV9-GFP mice when compared to sham control mice (Figures 3B,C). These data indicate that *cBin1* gene therapy can block, postpone, or even reverse the worsening cycles of HF progression in hearts with pre-existing HF, resulting in functional protection and better survival.

**Figure 4:**
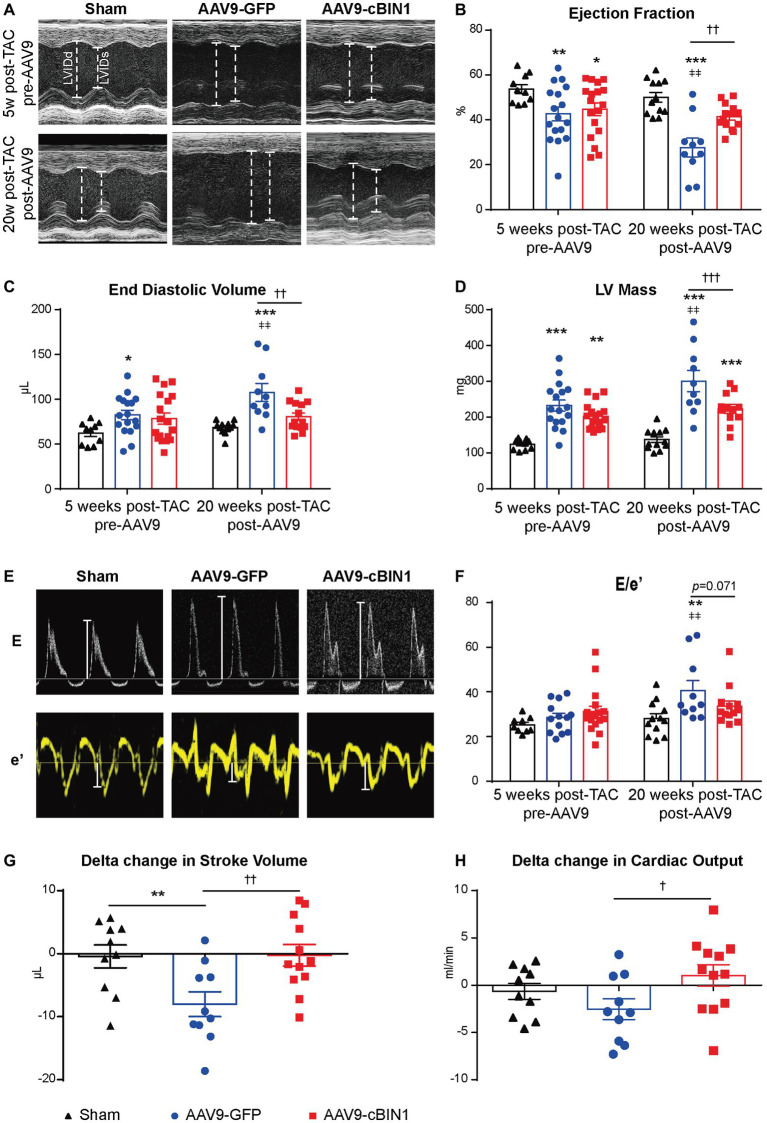
*cBin1* gene transfer preserves myocardial systolic and diastolic function in pressure overloaded hearts. **(A)** Representative left ventricular (LV) short axis M-mode images from each group (sham, AAV9-GFP, AAV9-cBIN1) at 5 weeks post-TAC (pre-AAV9 injection) and 20 weeks post-TAC (15 weeks post-AAV9 injection). Echocardiography-measured **(B)** left ventricle ejection fraction (EF), **(C)** end-diastolic volume, and **(D)** left ventricle mass at 5 (pre-AAV9) and 20 (post-AAV9) weeks post-TAC. **(E)** Representative mitral valve inflow pulsed wave (E) Doppler images (top) and tissue Doppler images of septal mitral valve annulus (e') (bottom) at 20 weeks post TAC (15 weeks post-AAV9 injection). **(F)** Quantification of E/e' from each group at 5 (pre-AAV9) and 20 (post-AAV9) weeks post-TAC. Delta changes from 5-week to 20-week in **(G)** stroke volume (SV) and **(H)** cardiac output (CO) of each mouse (ΔSV = SV_20w_–SV_5w_; ΔCO = CO_20w_–CO_5w_). Data are presented as mean ± SEM, and two-way ANOVA with Fisher’s LSD test was used for statistical analysis. ^*,**,***^p < 0.05, 0.01, 0.001 vs. sham; ^†,††,†††^*p* < 0.05, 0.01, 0.001 comparing between AAV9-GFP and -cBIN1 groups at each time point. ^ǂǂ^*p* < 0.01 when comparing pre‐ and post-AAV9 treatment in each group.

Echocardiography-measured myocardial functional parameters were further compared across groups both before and after AAV9 treatment (Table 1). At 20 weeks post-TAC, AAV9-GFP mice developed significant LV contractile dysfunction (EF reduction) and chamber dilation (EDV elevation; Figures 4A–C and Table 1), which were normalized by AAV9-cBIN1 treatment. The increase in LV Mass at 20 weeks post-TAC was significantly reduced in AAV9-cBIN1 treated mice when compared to AAV9-GFP group (Figure 4D). In AAV9-GFP treated mice, TAC surgery resulted in a progressive increase in E/e', indicating the onset of diastolic dysfunction. AAV9-cBIN1, on the other hand, effectively blocked progressive worsening of E/e', indicating exogenous cBIN1-mediated preservation of cardiac lusitropy. As a result, E/e' values of 20 weeks post-TAC cBIN1 hearts were maintained at their pre-AAV levels, which were not significantly different from E/e' values of sham control hearts but tended to be lower than those of AAV9-GFP hearts (*p* = 0.071) at 20 weeks post-TAC (Figures 4E,F). Furthermore, the observed delta reductions in stroke volume and cardiac output in AAV9-GFP group were also abolished in AAV9-cBIN1 treated group (Figures 4G,H). These results indicate that *cBin1* gene therapy preserves myocardial function when administered to mice with failing hearts.

**Figure 5:**
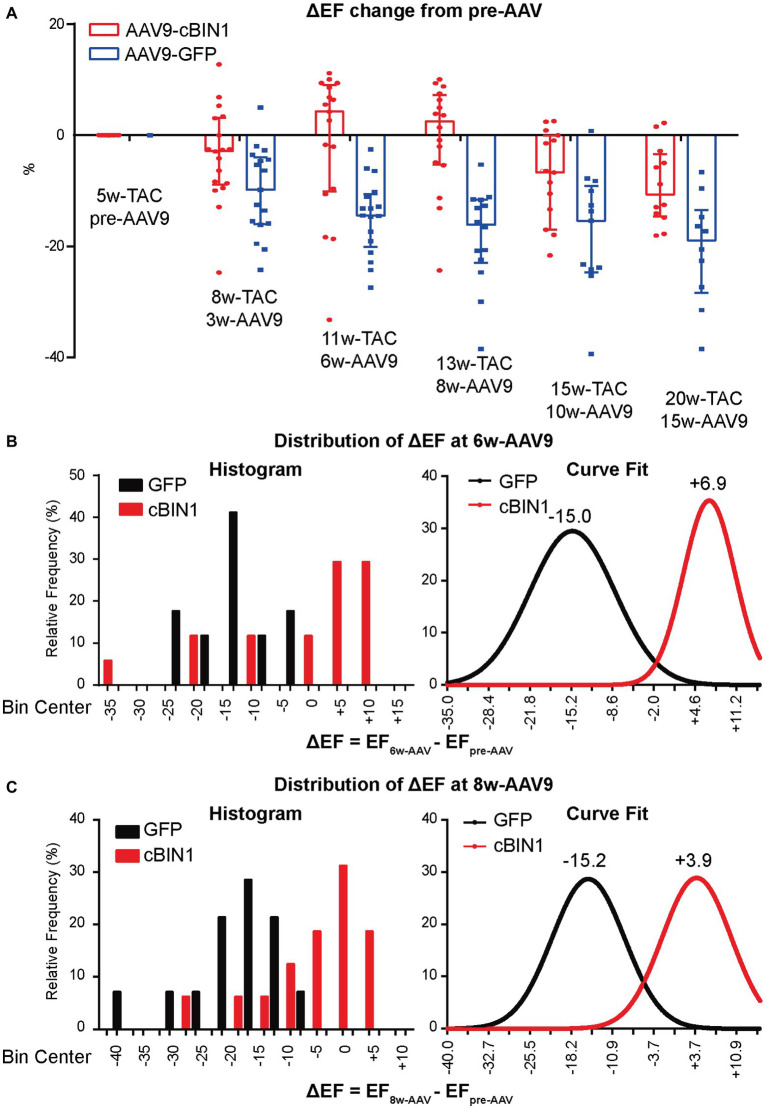
AAV9-cBIN1 post-treatment rescues EF in post-TAC mouse hearts. **(A)** Echocardiography monitored delta EF changes (ΔEF) from pre-AAV to 3, 6, 8, 10, and 15 weeks post AAV9 injection (correspondingly 8, 11, 13, 15, and 20 weeks post-TAC) in AAV9-CMV-GFP and -cBIN1 treating groups. Histogram distribution of ΔEF at **(B)** 6-week and **(C)** 8-week post AAV9 treatment with Gaussian distribution fitting curve. Data are presented as mean ± SEM.

To explore the progression of systolic dysfunction in post-TAC hearts after viral injection at Time 0 (pre-AAV9, 5 weeks post-TAC), the delta EF changes (ΔEF) from pre-AAV to 3, 6, 8, 10, and 15 weeks post-AAV9 injection (corresponding to 8, 11,13, 15, and 20 weeks post-TAC) were monitored by echocardiography (Figure 5A). Exogenous cBIN1-induced EF recovery peaked at 6–8 weeks post-AAV9 injection with continuous improvement of EF in the following weeks, whereas progressive EF reduction was noted in the AAV9-GFP group. The observed recovery of EF is demonstrated on the histogram distribution of ΔEF with Gaussian fitting (Figures 5B,C). AAV9-cBIN1 has a right-shifted histogram distribution of ΔEF when compared to AAV9-GFP group. For instance, at 6 weeks post-AAV9, there is a medium EF (%) reduction of −15.0 in AAV9-GFP group, while a medium recovery of +6.9 in EF (%) is observed in the AAV9-cBIN1 group. These data indicate that exogenous cBIN1, when administered at 5 weeks post-TAC, can improve myocardial systolic function in hearts with TAC-induced HF.

**Figure 6:**
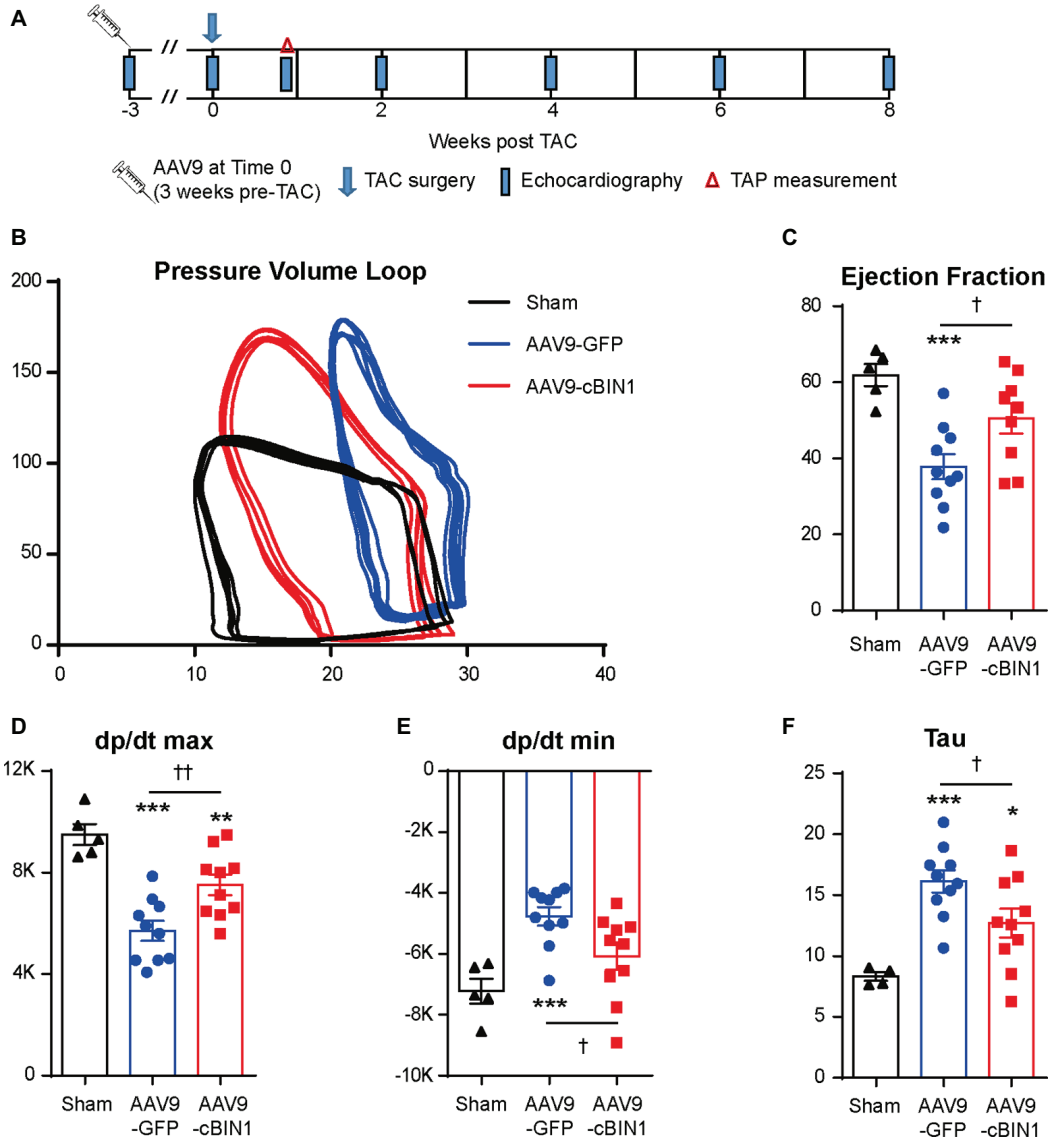
Exogenous cBIN1 pretreatment improves myocardial pressure-volume (PV) loops. **(A)** Schematic protocol: sham (*N* = 5) or TAC with pretreatment of AAV9-GFP (*N* = 10) or cBIN1 (*N* = 10) administered 3 weeks prior to TAC. Representative PV loop **(B)**, EF **(C)**, dp/dt max **(D)**, dp/dt min **(E)** and Tau **(F)** in sham, AAV9-GFP and -cBIN1 hearts at 8 weeks post TAC surgery. Data are presented as mean ± SEM, and one-way ANOVA with Fisher’s LSD test was used for statistical analysis. ^**,***^p < 0.01, 0.001 vs. sham; ^†,††^*p* < 0.05, 0.01 comparing between AAV9-GFP and AAV9-cBIN1 groups.

We recently reported that, in mice receiving AAV9-cBIN1 pretreatment (3 × 10^10^ vg at 3 weeks prior to TAC surgery; Figure 6A), the incidence of TAC-induced HF is significantly reduced with a resultant better HF-free survival at 8 weeks post-TAC (Liu et al., 2020). These data are consistent with the observed myocardial protection when AAV9 was administered after TAC surgery. To further establish the cardioprotective effect of exogenous-cBIN1 in the TAC mice, intracardiac hemodynamics were obtained in AAV9-pretreated mice using invasive PV loop recording. Figure 6B contains representative PV loops of sham, AAV9-GFP, and AAV9-cBIN1 pretreated hearts 8 weeks after TAC surgery (Figure 6B). The EF and the maximal rate of pressure change during systole (dp/dt max) were decreased in AAV9-GFP group, which were normalized by exogenous cBIN1 (Figures 6C,D). When exploring the effects on relaxation kinetics, we found that the maximal rate of pressure decay (dp/dt min) was decreased in AAV9-GFP group but was normalized by AAV9-cBIN1 (Figure 6E). The increased time constant for isovolumic relaxation (Tau) in AAV9-GFP group was also rescued by *cBin1* gene transfer (Figure 6F), indicating improved cardiac relaxation. Together, these data indicate that exogenous cBIN1 improves cardiac inotropy and lusitropy in pressure overloaded hearts.

**Figure 7:**
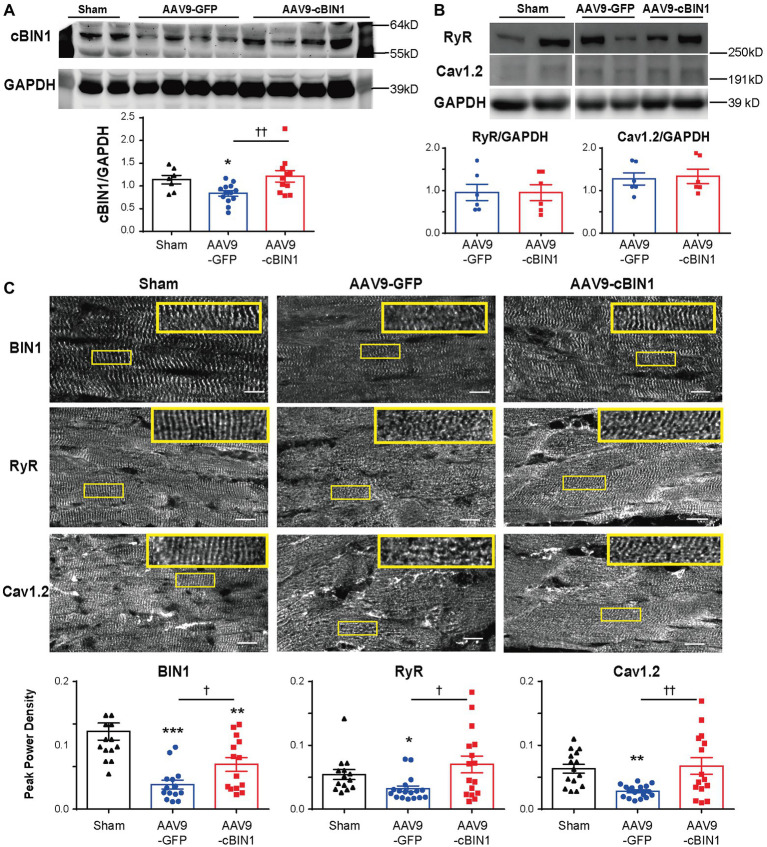
cBIN1-microdomain reduction in post-TAC hearts can be normalized with AAV9-cBIN1 pretreatment. Western blot of **(A)** cBIN1, **(B)** ryanodine receptor (RyR), and Cav1.2 from sham, AAV9-GFP, and AAV9-cBIN1 pretreated post-TAC heart lysates. Quantitation is included in the bar graph at the bottom (*n* = 8 hearts per group for cBIN1, *n* = 6 hearts per group for cBIN1). **(C)** Representative myocardial immunofluorescent spinning-disc confocal images of BIN1 labeling (anti-BAR domain; top panel), RyR (middle panel), and Cav1.2 (bottom panel) from sham, AAV9-GFP, and AAV9-cBIN1 pretreated post-TAC hearts. The insets include enlarged images of the corresponding boxes areas. Bottom row (from left to right): Peak power density of BIN1, RyR, and Cav1.2 distribution in sham, AAV9-GFP and cBIN1-pretreated hearts at 8 weeks post-TAC surgery (*n* = 15–20 images from five hearts per group). Data are presented as mean ± SEM, and one-way ANOVA with Fisher’s LSD test was used for statistical analysis. ^*,**,***^p < 0.05, 0.01, 0.001 vs. sham; ^†,††^*p* < 0.05, 0.01 comparing between AAV9-GFP and -cBIN1 groups.

We previously identified that cBIN1 creates t-tubule microdomains and organizes LTCC-RyR dyads for efficient and dynamic regulation of cardiac function and EC coupling (Hong and Shaw, 2017). More recently, we found that in sympathetic overdriven mouse hearts developing diastolic dysfunction, cBIN1-microdomains are disrupted and are rescued by AAV9-cBIN1. Here, we also explored the alterations in cardiac t-tubule cBIN1-microdomains and the effect of exogenous cBIN1 in post-TAC hearts. Western blotting (Figure 7A) identifies that myocardial cBIN1 protein is significantly reduced in mouse hearts 8 weeks after TAC (27% less than sham controls, *p* < 0.05), which is normalized by AAV9-cBIN1 pretreatment. Along with cBIN1 rescue, membrane labeling with Di-8-ANNEPs (Supplementary Figure 2) identifies that compared to sham cardiomyocytes, 8 weeks post-TAC cardiomyocytes have a significantly reduced t-tubule cBIN1-microdomain intensity, which is normalized in cardiomyocytes from mice with AAV9-cBIN1 pretreatment. We, next used immunofluorescent imaging to analyze the organization of cBIN1-microdomains and dyads at the myocyte level. Power spectrum analysis of BIN1 signal identifies that the well-organized t-tubule distribution of cBIN1 in sham myocardium is disrupted in post-TAC hearts, which is preserved with AAV9-cBIN1 pretreatment (Figure 7C). Although total LTCC and RyR protein levels are not significantly altered among groups by Western blotting (Figure 7B), myocardial distribution of LTCCs and RyRs (Figure 7C) becomes disorganized in post-TAC hearts, which is also significantly improved in hearts with AAV9-cBIN1 pretreatment (*p* < 0.05). These data indicate that exogenous cBIN1 normalizes TAC-induced reduction of myocardial cBIN1, resulting in preservation of cBIN1-scultped microdomains at t-tubules. *Via* normalizing t-tubule microdomains in pressure-overloaded hearts, cBIN1 replacement therapy thus reorganizes cardiac LTCC-RyR couplons required for beat-to-beat calcium cycling and efficient EC coupling.

## Discussion

In this study, we report that AAV9 virus transduced-exogenous cBIN1 in myocardium, applied after a reduction in EF, can improve cardiac function and limit further development of ventricular chamber dilation and HF in mice subjected to chronic pressure overload stress. The critical role of cBIN1 in regulating cardiac function is consistent with previous findings that cBIN1-organized t-tubule microdomains are required for normal dyad formation and function (Hong et al., 2010, 2012a,b, 2014; Hong and Shaw, 2017), and that reduced cBIN1 expression contributes to weakened calcium transients and impaired cardiac contractility in failing hearts (Hong et al., 2012b; Lyon et al., 2012; Caldwell et al., 2014).

Under continuous pressure overload, myocardial remodeling starts with an adaptive hypertrophic response followed by transitioning into maladaptive cardiac dilatation, leading to worsening HF (Liu et al., 2011; Zhang et al., 2016; Zhou et al., 2016). In previous studies, we identified that the administration of AAV9-cBIN1 prior to TAC surgery preserves myocardial systolic and diastolic function, indicating the efficacy of *cBin1* gene therapy in HF prevention. In the current study, we found that exogenous cBIN1 administration after TAC-induced HF blocks further disease progression and improves the overall survival with attenuated cardiac hypertrophy and lessened pulmonary edema in mice (Figures 2, 3). Furthermore, exogenous cBIN1 introduced by gene transfer improves myocardial remodeling and cardiac function as measured by echocardiography (Figures 4, 6). Most strikingly, mice with preexisting HF exhibited recovered EF following *cBin1* gene therapy (Figure 5), indicating the protective effect of exogenous cBIN1 may serve as a translatable treatment for patients with diagnosed preexisting structural remodeling and HF. Furthermore, consistent with results in isoproterenol chronically stressed mouse hearts (Liu et al., 2020), gene therapy-induced cBIN1 restoration in post-TAC hearts occurs at t-tubule microdomains and is capable of inducing the reorganization of LTCC-RyR couplons (Figure 7), indicating the observed therapeutic effect is mechanistically linked to normalization of impaired calcium handling in failing cardiomyocytes.

Recently, AAV-mediated gene therapy has been shown as a promising modality for the treatment of HF (Rincon et al., 2015; Bass-Stringer et al., 2018). There are currently several completed or ongoing clinical trials of HF gene therapies targeting various pathways such as the β-adrenergic system, Ca^2+^ cycling proteins, and cell death pathways, as well as homing stem cells (Tilemann et al., 2012). We recently found that targeting the calcium regulating microdomains at t-tubules can be effectively achieved by transducing the essential microdomain-organizing protein cBIN1 (Liu et al., 2020). By stabilizing t-tubule microdomains, cBIN1 preserves cytosolic calcium homeostasis and contributes to increasing systolic calcium release, improving diastolic reuptake, limiting SR leak for electrical stability maintenance, and preserving mitochondrial function to limit mitochondrial-associated cell death. Our data indicate that this microdomain-targeting approach may serve as a new therapeutic strategy with improved efficiency in functional preservation, improving overall HF survival. Furthermore, the observed cBIN1-mediated improvement in overall survival (Figure 2) is a possible combined effect from improved pump function and reduced arrhythmias, both of which are regulated by cBIN1-microdomains (Hong et al., 2012b, 2014; Hong and Shaw, 2017). How cBIN1 therapy affects arrhythmia burden in failing hearts will need further analysis using *in vivo* telemetry monitoring in future studies. In addition, since TAC-induced HF is associated with mitochondrial disorder-associated myocyte death (Thai et al., 2018), it remains interesting in future studies to explore whether cBIN1 replacement therapy can preserve mitochondrial function and limit mitochondrial-related cell death in failing hearts.

With regard to functional recovery, although EF changes monitored from the beginning of AAV9-cBIN1 treatment shows a peak recovery at week 6 post-AAV9 followed by descending therapeutic efficiency (Figure 5), the positive effect is maintained at 15-week post AAV9 injection as shown in Figure 4. These data indicate that even a single administration of AAV9-cBIN1 at a relatively low dose (3 × 10^10^ vg) is effective in preserving cardiac function. Whether multiple administrations of exogenous cBIN1 with increased dosage are needed to maximize its therapeutic effect remains to be tested. Nevertheless, our current data (Figures 4, 5) indicate that, for patients with existing HF, *cBin1* gene therapy could potentially break the worsening cycles of HF progression and result in functional recovery of failing hearts.

In conclusion, our study reveals a protective role of exogenous cBIN1 in mouse hearts with existing HF after subjected to pressure overload. For this proof-of-concept study, we used the AAV9 vector driven by the CMV promoter for gene delivery due to its consistent transduction efficiency and established cardiac tropism. Further experiments using *cBin1* packaged in AAV9 with a more efficient cardiac-specific promoter in mice and large mammals will be needed before clinical trials testing the efficacy and efficiency of *cBin1* gene therapy in HF patients. Future studies are also needed to explore the intracellular mechanism for cBIN1 in balancing calcium homeostasis among cytosolic microdomains at t-tubules, SR, and nearby mitochondria. Further understanding of the downstream targeting molecules and signaling pathways of cBIN1 will be needed as well for a better understanding of the interplay between *cBin1* gene therapy and HF pathophysiology.

## Data Availability Statement

The raw data supporting the conclusions of this article will be made available by the authors, without undue reservation.

## Ethics Statement

The animal study was reviewed and approved by the Cedars-Sinai Medical Center (CSMC) and University of Utah Institutional Animal Care and Use Committees (IACUC).

## Author Contributions

TH and RS contributed to conception and design the study. TH supervised the experiments. JL and SA were responsible for performing experiments and analyzing the data. KZ performed the PV loop experiments. JL and TH wrote the manuscript. TH and RS revised the manuscript. All authors read and approved the final manuscript.

### Conflict of Interest

The authors declare that the research was conducted in the absence of any commercial or financial relationships that could be construed as a potential conflict of interest.

## Acknowledgments

We are grateful to Tara Hitzeman, MPH for help with statistics.

## Supplementary Material

The Supplementary Material for this article can be found online at: https://www.frontiersin.org/articles/10.3389/fphys.2020.00708/full#supplementary-material.
