# Supplementary material for: Exogenous Cardiac Bridging Integrator 1 Benefits Mouse Hearts With Pre-existing Pressure Overload-Induced Heart Failure
Source: Front Physiol. 2020 Jun 24;11:708. doi: 10.3389/fphys.2020.00708 (PMC7327113; doi:10.3389/fphys.2020.00708)
Supplement: Supplementary file 1 [file Data_Sheet_1.PDF]

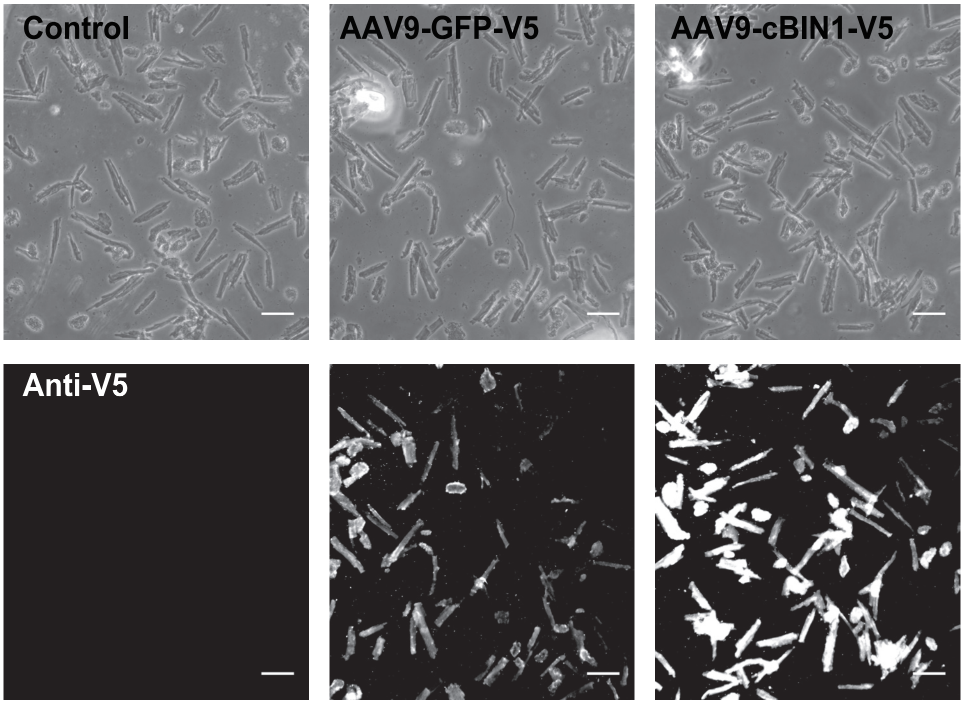

Supplemental Figure 1. AAV9-transduced exogenous GFP-V5 and cBIN1-V5 protein expression in mouse cardiomyocytes. Representative adult mouse ventricular cardiomyocyte images under transmission light (top) or widefield fluorescent light (rabbit anti-V5 labeling, bottom) from control (left), AAV9-GFP-V5 (middle), or AAV9-cBIN1-V5 (right) treated mice.

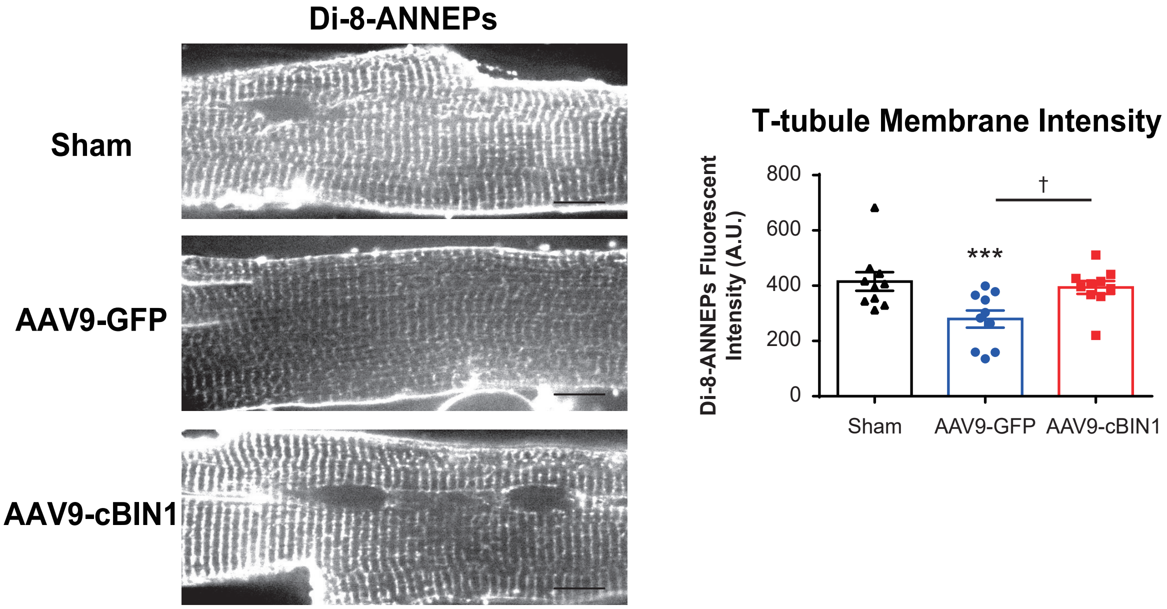

Supplemental Figure 2. AAV9-transduced exogenous cBIN1 normalizes cardiomyocyte t-tubule microfolds in post-TAC hearts.
Representative live-cell membrane labeling (Di-8-ANNEPs) images of cardiomyocytes freshly isolated from sham, AAv9-GFP and AAV9-cBIN1 treated post-TAC hearts. Quantification of t-tubule Di-8-ANNEPs intensity is included in the bar graph to the right (n=10 images from 5 hearts per group). All data are presented as mean ± SEM. Kruskal-Wallis with LSD post test was used for statistical analysis. ***, $p<0.001$ when comparing to Sham group; †, $p<0.05$ when comparing between AAV9-GFP and AAV9-cBIN1 groups.
